# A 4-week, lifestyle-integrated, home-based exercise training programme elicits improvements in physical function and lean mass in older men and women: a pilot study

**DOI:** 10.12688/f1000research.11894.2

**Published:** 2017-09-11

**Authors:** Jessica Cegielski, Matthew S. Brook, Jonathan I. Quinlan, Daniel J. Wilkinson, Kenneth Smith, Philip J. Atherton, Bethan E. Phillips

**Affiliations:** 1 MRC-ARUK Centre for Musculoskeletal Ageing Research, School of Medicine, University of Nottingham, Derby, UK

**Keywords:** home-based, exercise, muscle mass, muscle function

## Abstract

Background: Developing alternative exercise programmes that can alleviate certain barriers to exercise such as psychological, environmental or socio-economical barriers, but provide similar physiological benefits e.g. increases in muscle mass and strength, is of grave importance. This pilot study aimed to assess the efficacy of an unsupervised, 4-week, whole-body home-based exercise training (HBET) programme, incorporated into daily living activities, on skeletal muscle mass, power and strength.

Methods: Twelve healthy older volunteers (63±3 years, 7 men: 5 women, BMI: 29±1 kg/m²) carried out the 4-week “lifestyle-integrated” HBET of 8 exercises, 3x12 repetitions each, every day. Before and after HBET, a number of physical function tests were carried out: unilateral leg extension 1-RM (one- repetition maximum), MVC (maximal voluntary contraction) leg extension, lower leg muscle power (via Nottingham Power Rig), handgrip strength and SPPBT (short physical performance battery test). A D _3_-Creatine method was used for assessment of whole-body skeletal muscle mass, and ultrasound was used to measure the quadriceps cross-sectional area (CSA) and *vastus lateralis *muscle thickness.

Results: Four weeks HBET elicited significant (p<0.05) improvements in leg muscle power (276.7±38.5 vs. 323.4±43.4 W), maximal voluntary contraction (60°: 154.2±18.4 vs. 168.8±15.2 Nm, 90°: 152.1±10.5 vs. 159.1±11.4 Nm) and quadriceps CSA (57.5±5.4 vs. 59.0±5.3 cm ^2^), with a trend for an increase in leg strength (1-RM: 45.7±5.9 vs. 49.6±6.0 kg, P=0.08). This was despite there being no significant differences in whole-body skeletal muscle mass, as assessed via D _3_-Creatine.

Conclusions: This study demonstrates that increases in multiple aspects of muscle function can be achieved in older adults with just 4-weeks of “lifestyle-integrated” HBET, with a cost-effective means. This training mode may prove to be a beneficial alternative for maintaining and/or improving muscle mass and function in older adults.

## Introduction

Older age is associated with the loss of skeletal muscle mass. Termed *sarcopenia*, age-related muscle wasting is associated with loss of muscle strength ( *dynapenia*), increased morbidity, loss of independence and premature mortality ( Rudrappa *et al*., 2016). There are however proven means by which to offset these detrimental progressive declines. For example, structured and fully-supervised progressive resistance exercise training (RET) has been shown to improve both muscle mass ( Phillips *et al*., 2012) and function ( Liu & Latham, 2009).

Despite the established effects of RET on muscle mass and function, compliance is difficult to achieve, due to perceived (and real) socio-economic, psychological and environmental factors ( Zlot *et al*., 2006). Almost all current evidence for the efficacy of RET is based on 2–3 sessions each week, for up to 12 weeks, at a gym or with specialist equipment, reflecting current physical activity guidelines for adults of 2–3 days per week RET for each of the major muscle groups ( Bull *et al*., 2010). However, compliance to physical activity recommendations is poor, with recent data suggesting that they are only achieved by 38% of UK adults ( Department of Health, Physical Activity, 2011), a figure that is lesser still in older adults ( Jefferis *et al*., 2014). As a result, home-based exercise training (HBET) programmes have been suggested as an alternative to overcome some of the barriers associated with poor compliance to exercise ( Hong *et al*., 2017; Silveira *et al*., 2013), whilst also providing a platform to develop the benefits of RET for (pre-) sarcopenic individuals ( Maruya *et al*., 2016).

The majority of HBET studies to date, have focussed on pre- or rehabilitation for specific clinical groups ( Bassett & Prapavessis, 2007; Chang *et al*., 2017). A small number of studies have specifically investigated the efficacy of HBET on skeletal muscle function in the elderly, although the focus of these studies has mainly been on lower limb exercises to improve walking and balance ( Ito *et al*., 2015; Yamauchi *et al*., 2009) in relation to fall and fracture prevention. Other studies in older adults have incorporated the use of specialist aids e.g. dumbbells ( Plotnikoff *et al*., 2010), elastic bands ( Jette *et al*., 1999; Ribeiro *et al*., 2009) or ankle weights ( Gardner *et al*., 2001) to provide resistance for HBET; rather than attempting to incorporate exercise into tasks of daily living, as is done herein. To date, no studies have attempted to investigate the efficacy of incorporating whole-body exercise into activities of daily living on muscle mass and function in older adults. Consequently, in this pilot study we aimed to assess the effects of a 4-week, progressive, whole-body HBET fully integrated into activities of daily living, on muscle mass and function in healthy older individuals. We also aimed to utilise a recently re-introduced ( Clark *et al*., 2014) and underexploited creatine method to estimate whole-body muscle mass in humans.

## Materials and methods

### Ethical statement and recruitment of study participants

Twelve healthy older male and female volunteers (63±3 y, 7 men: 5 women, BMI: 29±1 kg/m ^2^) with no previous chronic disease history were recruited via an advertisement on a local radio station. Before enrolment into the study, all volunteers had an assessment of previous medical history and an ECG to check for any subclinical arrhythmias. The ECG’s were approved by a clinically qualified physician. All volunteers provided written informed consent prior to the start of this study. This study was approved by The University of Nottingham Medical School Ethics Committee (B28102015 SoMS GEM BBC) and conformed to The Declaration of Helsinki.

### Study procedures

**Figure 1.**
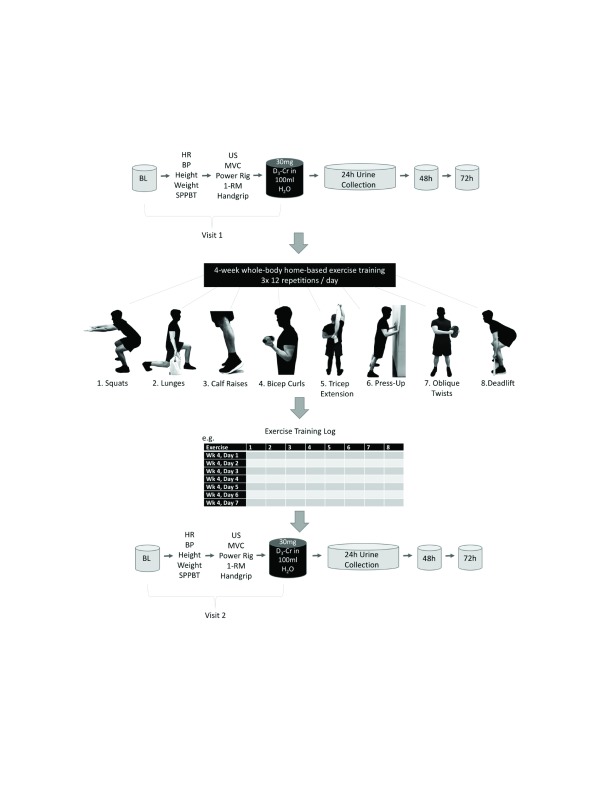
Study schematic displaying the two visits before and after home-based exercise training (HBET). BL: baseline urine sample; HR: resting heart rate; BP: resting blood pressure; SPPBT: short physical performance battery test; US: leg ultrasound; MVC: maximum voluntary contraction; 1-RM: leg extension 1-repetition maximum; D _3_-Cr: D _3_-Creatine for whole-body muscle mass assessment; 48 and 72h represent the time of urine samples post-tracer consumption.

The study involved two visits by the study participants, before and after the 4-week HBET ( Figure 1), to the University of Nottingham Medical School, at the Royal Derby Hospital. All assessments at these visits were conducted by researchers from the MRC-ARUK Centre for Musculoskeletal Ageing Research. At each visit, volunteers provided a baseline urine sample for the muscle mass assessment by D _3_-Creatine before undergoing measures of height, weight (Marsden Weighing Group, UK), resting heart rate and blood pressure (Omron M5-1 digital BP monitor, Omron, UK). Each volunteer had the thickness, fascicle length ( *L _f_*) and pennation angle ( *θ*_*pen*_) of their *vastus lateralis* muscle *,* and the cross-sectional area (CSA) of their quadriceps measured by ultrasound (Mylab 70, Esaote Biomedica, Genova, Italy), using the protocol described by Franchi *et al*. (2014). Volunteers then completed a number of muscle function assessments:

• maximal voluntary contraction (MVC) for seated leg extension and flexion using an isokinetic dynamometer (Isocom; Isokinetic Technologies, Eurokinetics, UK),
• leg extension 1-repetition maximum (1-RM; ISO leg extension, Leisure Lines Ltd., GB),
• leg power (Nottingham Power Rig; Bassey & Short, 1990),
• handgrip strength (Grip D 5401; Takei Scientific Instruments Co. Ltd., Japan); and
• a short physical performance battery test (SPPBT; Guralnik *et al*., 1994).

MVC was measured at two knee angles (60 and 90°), whilst isokinetic (eccentric/concentric) contractions were measured over a 70° range (20–90°) at three different speeds (60, 180 and 240°/sec). For both measures, horizontal seated leg extension was set at 0°. Following the muscle function assessments, volunteers ingested 30mg of D _3_-Creatine dissolved in 100ml H _2_O for muscle mass assessment, before providing a pooled 24h urine collection and single urine samples at 48 and 72 h post consumption ( Clark *et al*., 2014).

### Home-based exercise training programme (HBET)

Volunteers were required to complete 3 sets of 12 repetitions across 8 different exercises, every day, for 4 weeks. Each exercise was designed to be easily incorporated into activities of daily living to minimise the time-commitment required (e.g. bicep curls whilst cooking, see Figure 1). In total the exercise programme consisted of: squats, lunges, calf raises, bicep curls, triceps extensions, semi-incline press-ups, oblique twists and deadlifts. Participants were instructed to take 2 seconds to perform each phase (eccentric and concentric) of each exercise and to hold for 2 seconds at the mid-point of each exercise. Progression via increased resistance or time-under-tension was encouraged once the full complement of repetitions could be completed. Compliance to exercise was measured by self-report (see self-report log in Figure 1). An exercise would only be marked as complete if 3 sets of 12 repetitions had been performed within a day.

### Measurement of whole-body muscle mass using D _3_-creatine

Using a creatine-tracer measure of muscle mass ( Clark *et al*., 2014), D _3_-creatine and D _3_-creatinine enrichment, plus unlabelled creatine and creatinine in the urine was measured using high-performance liquid chromatography/mass spectrometry (HPLC/MS), as detailed in ( Stimpson *et al*., 2012). Total muscle mass was calculated using the following equation:

 Total muscle mass =(MWUnlabelledMWlabelled)×(Amount of D3−Cr dosed(g)−Amount of D3−Cr excreted (g))(mean steady−state D−3Creatine enrichment ratio÷4.3(g/kg)

                                                                                                                                         ( Clark *et al*., 2014),

where *MW _Unlabelled_* and *MW _labelled_* represents the molecular weights of both unlabelled and labelled creatine, respectively. The estimated creatine pool size is then divided by 4.3 g/kg, which reflects the concentration of creatine in wet muscle.

### Statistical analysis

Descriptive statistics were carried out on all datasets and checked for a normal distribution using the Shapiro-Wilk normality test. Datasets were analysed using paired Student’s t test to assess the effect of HBET, with GraphPad Prism Software v5 (La Jolla, CA, USA). All data is presented as mean ±SEM. Significance was set at P<0.05.

## Results

### Compliance, body weight and cardiovascular parameters

Using data from the self-report training logs, overall compliance to the HBET was 87.3±3.0% over the 4-week period of training. Adherence to each individual exercise was >86%, with the calf-raises being the most completed exercise (91%). No significant changes were found following HBET for body weight (79.3±4.7 vs. 78.9±4.6 kg), resting heart rate (71.9±3.1 vs. 71.4±3.9 bpm) or blood pressure (systolic: 144.4±3.4 vs. 140.7±4.2, diastolic: 92.2±2.5 vs. 89.0±2.4; average of 3 measurements).

### Muscle function

**Figure 2.**
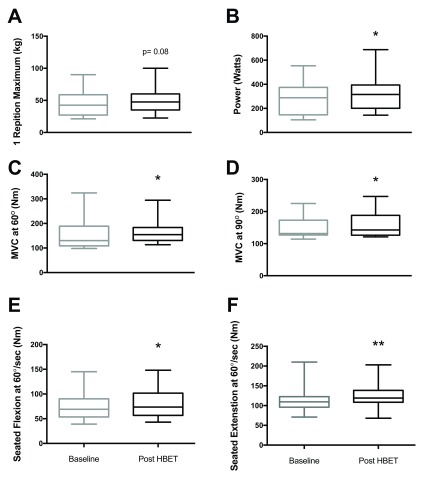
Muscle (leg) function assessments, before and after home-based exercise training (HBET). **A**) 1-RM leg extension (45.7±5.9 vs. 49.6±6.0 kg, P=0.08), **B**) Leg power via Nottingham Power Rig (276.7±38.5 vs. 323.4±43.4 W), **C**) MVC at 60° (154.2±18.4 vs. 168.8±15.2 Nm), **D**) MVC at 90° (152.1±10.5 vs. 159.1±11.4 Nm), **E**) Seated leg flexion at 60°/sec (74.7±8.3 vs. 80.8±8.6 Nm), **F**) Seated leg extension at 60°/sec (114.9±10.2 vs. 123.8±9.4 Nm). Baseline vs. post-HBET (mean ± SEM), n=12, *=p<0.05; **=p<0.01. MVC: Maximal voluntary contraction.

HBET did not elicit significant changes in leg extension 1-RM, handgrip strength or SPPBT; there was however, a trend for an increase in leg extension 1-RM (45.7±5.9 vs. 49.6±6.0 kg, P=0.08; Figure 2A). HBET did elicit significant increases in leg power (276.7±38.5 vs. 323.4±43.4 W, P<0.05; Figure 2B) and MVC at 60° (154.2±18.4 vs. 168.8±15.2 Nm, P<0.05; Figure 2C) and 90° (152.1±10.5 vs. 159.1±11.4 Nm, P<0.05; Figure 2D). There were also significant increases in peak torque recorded at 60°/sec during both seated flexion (74.7±8.3 vs. 80.8±8.6 Nm, P<0.05; Figure 2E) and extension (114.9±10.2 vs. 123.8±9.4 Nm, P<0.01; Figure 2F) after HBET.

### Muscle architecture

**Figure 3.**
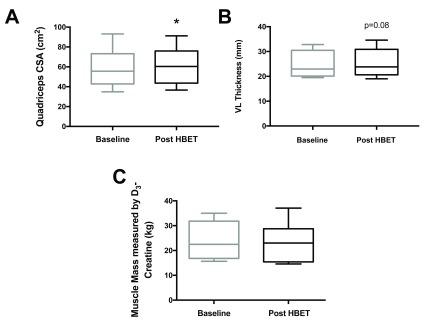
**A**) quadriceps cross-sectional area (CSA; 57.5±5.4 vs. 59.0 ± 5.3 cm ^2^); **B**) *m. vastus lateralis* (VL) thickness (25.0±1.6 vs. 25.5±1.7 mm, P=0.08); **C**) whole-body muscle mass, before and after home-based exercise training (HBET) (23.9±2.1 vs. 22.8±2.2 kg). (mean ± SEM), *=p<0.05, baseline vs. post-HBET.

CSA of the thigh at 50% of thigh length significantly increased (57.5±5.4 vs. 59.0±5.3 cm ^2^, P<0.05; Figure 3A) after HBET. However, there was no significant increase in muscle thickness, fascicle length ( *L _f_*) or pennation angle ( *θ*_*pen*_) of the *m. vastus lateralis*. There was a trend for an increase in muscle thickness (25.0±1.6 vs. 25.5±1.7 mm, P=0.08; Figure 3B) after HBET.

### Whole-body muscle mass measured by D _3_-Creatine

Corresponding with no significant change in body weight, there was no significant difference in whole-body muscle mass as estimated by D _3_-Creatine ( Figure 3C) after HBET (23.9±2.1 vs. 22.8±2.2 kg).

Raw data supporting the findings in this study Sheet 1: Maximum Voluntary Contraction (MVC) at 60° and 90°, pre- and post 4-weeks of home-based whole-body exercise training. Sheet 2: Seated leg extension measures at 60, 180 and 240 deg/sec pre- and post 4-weeks of home-based whole-body exercise training. Sheet 3: Seated leg flexion at 60, 180 and 240 deg/sec pre- and post- 4-weeks of home-based whole-body exercise training. Sheet 4: Handgrip strength, single-leg 1-RM leg extension, SPPBT (short physical performance battery tests) and leg muscle power, measured pre- and post- 4-weeks of home-based whole-body exercise training. Sheet 5: Participant characteristics. Heart rate (HR), systolic and diastolic blood pressure (BP) and body weight, pre- and post- 4-weeks of whole-body home-based exercise training. Sheet 6: Quadriceps cross-sectional area (CSA), vastus lateralis muscle thickness, fascicle length and pennation angle measured by ultrasound pre- and post- 4-weeks of whole-body home-based exercise training. Sheet 7: Muscle mass measured by using D _3_-creatine, pre- and post- 4 weeks of whole-body home-based exercise training. Click here for additional data file. Copyright: © 2017 Cegielski J et al. 2017 Data associated with the article are available under the terms of the Creative Commons Zero "No rights reserved" data waiver (CC0 1.0 Public domain dedication).

## Discussion

In this pilot study, we show that lifestyle-integrated, whole-body HBET has the potential to meaningfully improve muscle function in older adults. We have shown that this training mode can increase leg power by 16.9% in just 4-weeks. Although lower than the 37% power increase demonstrated in a solely lower-extremity focused HBET programme, this study was conducted over an 8-week period ( DeBolt & McCubbin, 2004), suggesting that if the magnitude of increase was to continue at a constant rate, our training mode may elicit similar improvements. Additionally, we demonstrate increases in muscle strength (via MVC at 60°) similar to that observed in response to a 6-week, fully-supervised, gym-based training study ( Brook *et al*., 2016). Furthermore, despite no significant increase, there was a positive trend in the leg extension 1-RM, similar to that seen in an 8-week HBET study by Zion *et al*. (2003). Thus, while muscle function adaptations to HBET were numerically lesser compared to longer-term structured exercise regimens (some requiring specialist equipment), our data support the notion that just 4-weeks of a ‘lifestyle-integrated’ HBET meaningfully improves muscle function.

Despite these positive results, no changes in handgrip strength were found, a similar result to Nelson *et al*. (2004), who implemented a 6-month home-based whole-body exercise programme in 70 male and female elderly individuals. This may be due to a lack of exercises specifically targeting the forearm flexors. Indeed, although it is well-established that extant grip strength represents a biomarker of muscle function in older adults ( Bassey & Harries, 1993), our data suggests that it is an insensitive biomarker for important functional and mass gains in other muscle groups (e.g. the legs) following certain exercise interventions.

Importantly, increases in thigh CSA (and a trend towards increased muscle thickness), which may partly explain the functional improvements, were observed. Measures of thigh CSA by ultrasound have been positively correlated with those by MRI ( Reeves *et al*., 2004), therefore suggesting (leg) muscle hypertrophy in this study. However, since this occurred in the absence of detectable changes in whole-body muscle mass as measured by D _3_-Creatine, we speculate i) that preferential hypertrophy of leg muscles occurred, but remained undetectable on a whole-body level; or ii) that measurement of whole-body muscle mass was below the detection threshold for the D _3_-creatine method.

We acknowledge limitations to our study design. The use of validated physical activity monitors (e.g. Actiheart, CamNtech, Cambridge, UK) to assess compliance to exercise would have provided greater information on the adoption of, and adherence to our exercise programme. Similarly, the use of interactive technologies (e.g. training applications for tablets/smartphones) for motivation and/or instruction may have improved adherence ( Silveira *et al*., 2013). The lack of improvements in SPPBT were likely a result of our volunteers achieving optimal scores before the intervention.

To conclude, our findings demonstrate that significant increases in muscle mass, maximal voluntary contraction, maximal power and isokinetic strength can be achieved with just 4-weeks of unsupervised, lifestyle-integrated HBET in older adults. These improvements were achieved via cost-effective means, without the requirement for specialist equipment, facilities or supervision ( DeBolt & McCubbin, 2004; Jette *et al*., 1996; Zion *et al*., 2003). With high compliance and beneficial physiological effects in only a 4-week period, this study highlights the potential for lifestyle-integrated HBET to improve skeletal muscle ‘health’ in older adults. As increases in muscle mass and strength are positively correlated with overall physical function ( Liu & Latham, 2009), this novel training mode may be of particular benefit to older frail/(pre-) sarcopenic adults.

## Data availability

The data referenced by this article are under copyright with the following copyright statement: Copyright: © 2017 Cegielski J et al.

Data associated with the article are available under the terms of the Creative Commons Zero "No rights reserved" data waiver (CC0 1.0 Public domain dedication). 

**Dataset 1: Raw data supporting the findings in this study.**

**Sheet** ***1: Maximum Voluntary Contraction (MVC) at 60° and 90°, pre- and post 4-weeks of home-based whole-body exercise training.***

**Sheet 2: Seated leg extension measures at 60, 180 and 240 deg/sec pre- and post 4-weeks of home-based whole-body exercise training**

**Sheet 3: Seated leg flexion at 60, 180 and 240 deg/sec pre- and post- 4-weeks of home-based whole-body exercise training**

**Sheet 4: Handgrip strength, single-leg 1-RM leg extension, SPPBT (short physical performance battery tests) and leg muscle power, measured pre- and post- 4-weeks of home-based whole-body exercise training**

**Sheet 5: Participant characteristics. Heart rate (HR), systolic and diastolic blood pressure (BP) and body weight, pre- and post- 4-weeks of whole-body home-based exercise training.**

**Sheet 6: Quadriceps cross-sectional area (CSA), vastus lateralis muscle thickness, fascicle length and pennation angle measured by ultrasound pre- and post- 4-weeks of whole-body home-based exercise training.**

**Sheet 7: Muscle mass measured by using D _3_-creatine, pre- and post- 4 weeks of whole-body home-based exercise training.**

DOI, 10.5256/f1000research.11894.d170288 ( Cegielski *et al*., 2017).
