# Supplementary material for: A 4-week, lifestyle-integrated, home-based exercise training programme elicits improvements in physical function and lean mass in older men and women: a pilot study
Source: F1000Res. 2017 Sep 11;6:1235. Originally published 2017 Jul 26. [Version 2] doi: 10.12688/f1000research.11894.2 (PMC5680536; doi:10.12688/f1000research.11894.2)
Supplement: Peer review history

# Peer review: Referee response for version 2

**DOI:** 10.5256/f1000research.13649.r27145

**Published:** 2017-11-08

**Authors:** Philip Hennis

**Affiliations:** 1 Nottingham Trent University, Nottingham, UK

This paper is well written and reports findings of an interesting pilot study with implications to clinical practice. Some revisions are suggested below:

 Introduction:

 A clear rationale for the study was described which resulted in a clear aim. I would remove the last sentence of the introduction regarding the aim to use the creatine method of assessing whole body muscle mass. This sentence detracts from the main study aim and the actual purpose of the study.

 Methods:

 The exercise intervention and the outcome measurements are described clearly and could be replicated. More information should be included about the participants and how they were recruited. Did the radio advertisement include specific information about who should respond? Were strict inclusion and exclusion criteria applied? The reader needs this information to fully interpret the results. In particular, participants' activity levels preceding the study will impact the reader's interpretation of the adherence rate and changes in all the outcome variables. 

 Results:

 No revisions suggested. 

 Discussion:

 A large part of your rationale for home based exercise was greater adherence to the exercise regime and this was measured in the study. As such, a paragraph is required in the discussion section comparing adherence in the current study with those of other studies using home, gym or hospital based training programs. 

 The limitations paragraph should acknowledge that this study did not use a control group for comparison and the implications of this on drawing strong conclusions.

I have read this submission. I believe that I have an appropriate level of expertise to confirm that it is of an acceptable scientific standard.

# Peer review: Referee response for version 1

**DOI:** 10.5256/f1000research.12852.r24550

**Published:** 2017-08-08

**Authors:** Stuart Gray

**Affiliations:** 1 Institute of Cardiovascular and Medical Sciences, University of Glasgow, Glasgow, UK

The authors have investigated the effects of HBET on muscle function. This is an important advance as resistance exercise is known to be of benefit to health and yet (due to reasons mentioned in the paper) it is performed by very few people. I have a few comments on this but overall the manuscript is well written and the data clearly presented.

 1) I would like to see a bit more detail on exactly how these exercise were integrated into daily life, e.g. what did you tell the participants to do exactly.

 2) The authors mention that it may be that the changes in whole body muscle mass may have been below the detection level of the d3-creatine method - could the authors elaborate and provide details. 

 3) May be worth citing https://www.ncbi.nlm.nih.gov/pubmed/24903908 ^1^ in the discussion about the lack of change in grip strength.

 4) I think it would be useful to highlight in the discussion (as you mentioned briefly when discussing the SPPBT results) that these participants were not that old and were very healthy.

I have read this submission. I believe that I have an appropriate level of expertise to confirm that it is of an acceptable scientific standard.

## References

[1] TielandMVerdijkLBde GrootLCvan LoonLJ: Handgrip strength does not represent an appropriate measure to evaluate changes in muscle strength during an exercise intervention program in frail older people. *Int J Sport Nutr Exerc Metab*.2015;25(1) : 10.1123/ijsnem.2013-0123 27-36 10.1123/ijsnem.2013-0123 24903908
